# Supplementary material for: Phenotypic consequences of RNA polymerase dysregulation in Escherichia coli
Source: Nucleic Acids Res. 2017 Aug 23;45(19):11131–43. doi: 10.1093/nar/gkx733 (PMC5737641; doi:10.1093/nar/gkx733)

SUPPLEMENTARY FIGURE LEGENDS

Figure S1. (A) Graphs showing (log_{10} of the optical density (OD_{600nm}) as function of time (hour)) of a culture of *E. coli* MC1061 cells containing pJ404 or pJ404:Gp2 (left) and pBAD33 or pBAD33:Gp2 (right). The arrow indicates when L-arabinose was added to the culture. **(B)** As in (A) but the experiment was conducted with *E. coli* MC1061 cells containing pBAD33 and pBAD:Gp2(R56E). **(C)** As in (A) but the experiment was conducted with *E. coli* BW25113 cells containing pBAD or pBAD33. **(D)** Graphs showing (log_{10} of the optical density (OD_{600nm}) as function of time (hour)) of a culture of *E. coli* MC1061 cells containing pBAD or pBAD:Gp2 where either one (top) or two doses (bottom) of L-arabinose was added at the time points indicted by the arrow (see text for details). The arrows indicate when L-arabinose was added to the culture. **(E)** Graphs showing (log_{10} of the optical density (OD_{600nm}) as function of time (hour)) of a culture of *E. coli* MC1061 cells containing pBAD or pBAD:Gp2 in which both plasmids were isolated at t=8 hours (top graph), transformed into fresh *E. coli* MC1061 cells and grown again as in the top graph in fresh growth medium - shown in the bottom graph (see text for details). The arrows indicate when L-arabinose was added to the culture. **(F)** Graphs showing (log_{10} of the optical density (OD_{600nm}) as function of time (hour)) of a culture of *E. coli* MC1061 cells containing pBAD or pBAD:Gp2 in which the cells from both cultures were harvested at t=12 (top graph), extensively washed and re-inoculated into fresh growth medium (bottom graph). The arrows indicate when L-arabinose was added to the culture.

Figure S2. (A) Fluorescence microscopy images of cytological profiles of *E. coli* cells containing MC1061/pBAD and MC1061/pBAD:Gp2 before and after treatment

with rifamycin. **(B)** Plot of the intensity of DNA staining (DAPI) in and the recovery of the DNA staining over the course of the experiment shown in Figure 1F (only selected time points are shown). **(C)** The volcano plots show distribution of all differentially expressed genes with $\log_2$ fold change > 2 in phase C compared to phase B. The number of genes up- and down-regulated at the different time points is shown on the right.

Figure S3. A bar chart showing the $\log_2$ fold change of 54 small non-coding RNA genes as $\log_2$ fold change > 2 at t=0.5, t=3 (phase B) and t=8 (phase C) relative to gene expression at t=0. The Hfq associated small non-coding RNAs are indicated with red asterisks.

Figure S4. A schematic showing the experiment done for determining the survival of *E. coli* cells containing MC1061/pBAD and MC1061/pBAD:Gp2 at t=3 (phase B) and t=8 (phase C) following antibiotic or stress exposure (see text and Materials and Methods for details).

Figure S5. Graph showing ($\log_{10}$ of the optical density (OD_{600nm}) as function of time (hour)) of a culture of *E. coli* MC1061 cells containing pBAD:Gp2. The arrow indicates when different concentration of L-arabinose was added to the culture to induce Gp2 expression.

Figure S6. **(A)** Heat map showing expression pattern of 127 genes of the σ^{54} regulon (as described in (27)) at t=0.5, t=3 and t=8 relative to t=0. **(B)** Graph showing ($\log_{10}$ of the optical density (OD_{600nm}) as function of time (hour)) of a culture of *E. coli*

BW25113 and BW25113:Δ*rpoN* cells containing pBAD or pBAD:Gp2. The arrow indicates when L-arabinose was added to the culture.

Supplementary Figure 1.

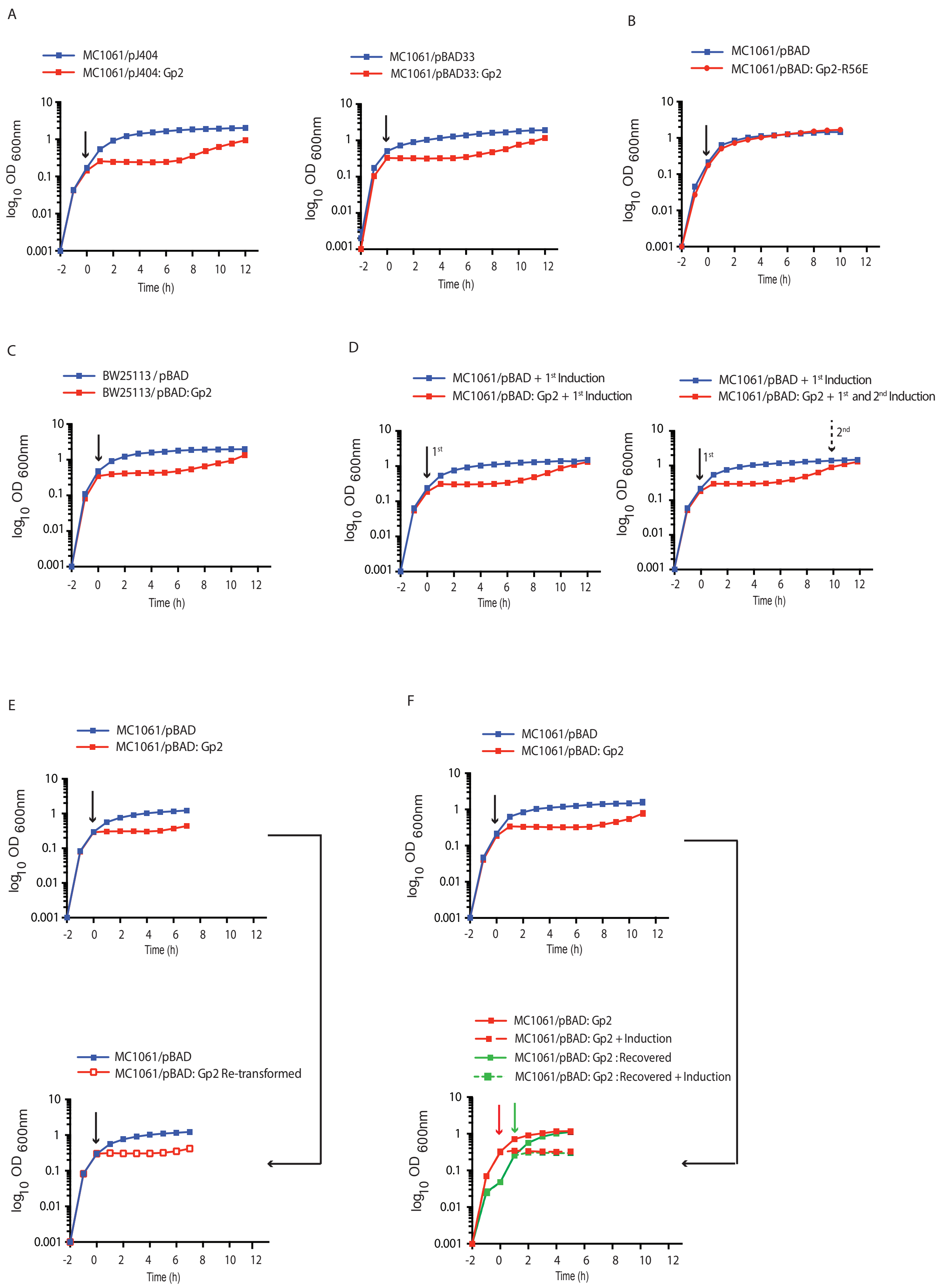

Supplementary Figure 2.

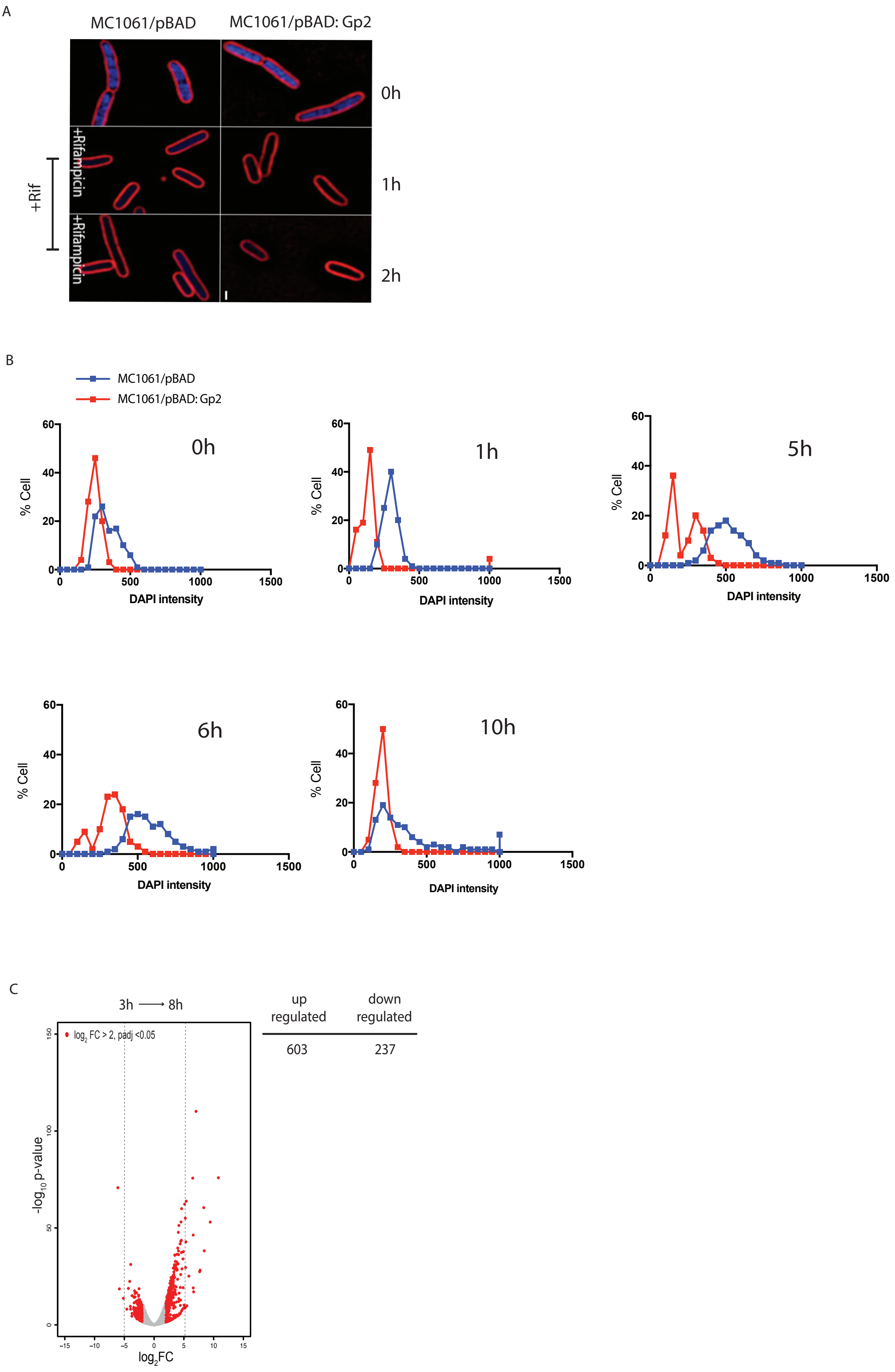

Supplementary Figure 3.

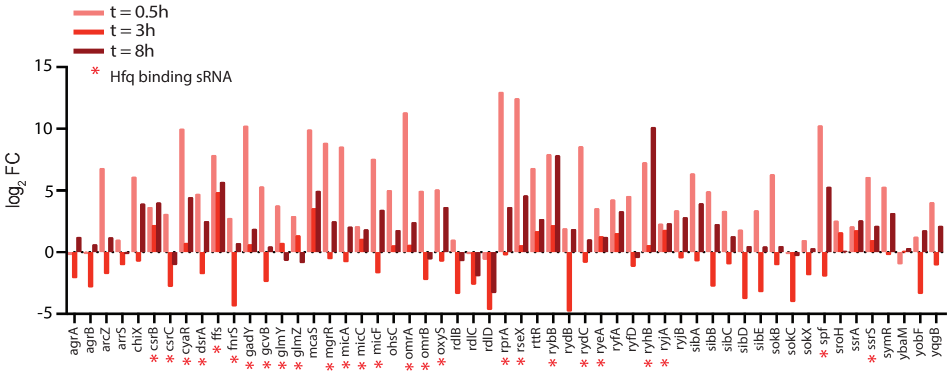

Supplementary Figure 4.

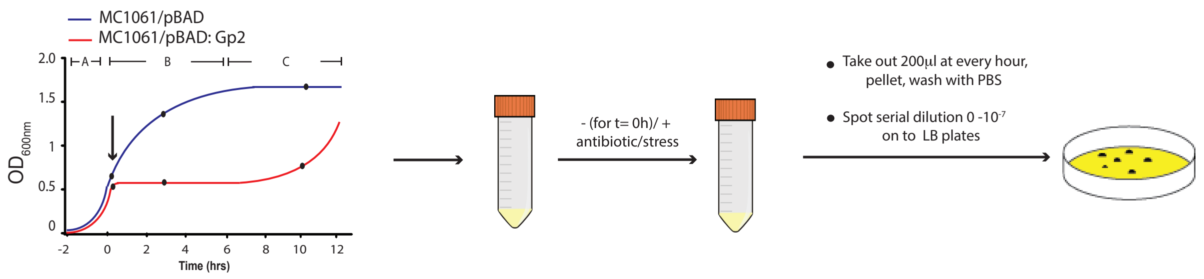

Supplementary Figure 5.

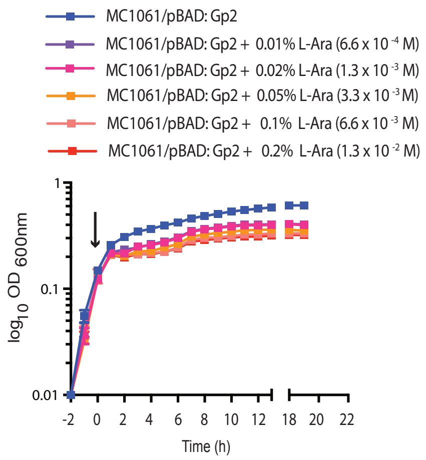

Supplementary Figure 6.

A

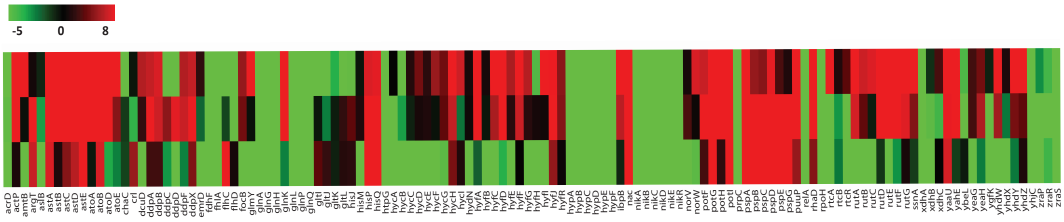

	up regulated	down regulated
t = 0.5h	41 (32%)	15(11%)
t = 3h	36 (28%)	22 (17%)
t = 8h	14 (11%)	23 (18%)

B

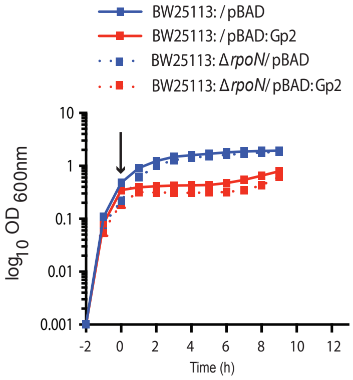

Supplement: Supplementary Data [file gkx733_supp.pdf]
